# Gastrointestinal consequences of lipopolysaccharide-induced lung inflammation

**DOI:** 10.1007/s00011-022-01657-0

**Published:** 2022-11-02

**Authors:** Rachel M. McQuade, Methma Bandara, Shanti Diwakarla, Lauren Sahakian, Myat Noe Han, Maryam Al Thaalibi, Madeleine R. Di Natale, Marsha Tan, Kiera H. Harwood, Elena K. Schneider-Futschik, Andrew Jarnicki

**Affiliations:** 1 Gut-Axis Injury and Repair Laboratory, Department of Medicine, Western Health, Western Centre for Health, Research and Education, The University of Melbourne, Sunshine Hospital, 176 Furlong Road, St Albans, Melbourne, VIC 3021 Australia; 2 Australian Institute for Musculoskeletal Science (AIMSS), Melbourne University, Melbourne, VIC 3021 Australia; 3 The Florey Institute of Neuroscience and Mental Health, Parkville, VIC 3010 Australia; 4 College of Health & Biomedicine, Victoria University, Melbourne, VIC Australia; 5 Department of Anatomy & Physiology, University of Melbourne, Parkville, VIC Australia; 6 Cystic Fibrosis Pharmacology Laboratory, Department of Biochemistry & Pharmacology, Melbourne University, Melbourne, VIC 3021 Australia; 7 Lung Health Research Centre, Department of Biochemistry & Pharmacology, Melbourne University, Melbourne, VIC 3021 Australia

**Keywords:** Lung inflammation, Gastrointestinal inflammation, Enteric neurons, Intestinal barrier, Gut-lung axis

## Abstract

**Background:**

Respiratory inflammation is the body’s response to lung infection, trauma or hypersensitivity and is often accompanied by comorbidities, including gastrointestinal (GI) symptoms. Why respiratory inflammation is accompanied by GI dysfunction remains unclear. Here, we investigate the effect of lipopolysaccharide (LPS)-induced lung inflammation on intestinal barrier integrity, tight-junctions, enteric neurons and inflammatory marker expression.

**Methods:**

Female C57bl/6 mice (6–8 weeks) were intratracheally administered LPS (5 µg) or sterile saline, and assessed after either 24 or 72 h. Total and differential cell counts in bronchoalveolar lavage fluid (BALF) were used to evaluate lung inflammation. Intestinal barrier integrity was assessed via cross sectional immunohistochemistry of tight junction markers claudin-1, claudin-4 and EpCAM. Changes in the enteric nervous system (ENS) and inflammation in the intestine were quantified immunohistochemically using neuronal markers Hu + and nNOS, glial markers GFAP and S100β and pan leukocyte marker CD45.

**Results:**

Intratracheal LPS significantly increased the number of neutrophils in BALF at 24 and 72 h. These changes were associated with an increase in CD45 + cells in the ileal mucosa at 24 and 72 h, increased goblet cell expression at 24 h, and increased expression of EpCAM at 72 h. LPS had no effect on the expression of GFAP, S100β, nor the number of Hu + neurons or proportion of nNOS neurons in the myenteric plexus.

**Conclusions:**

Intratracheal LPS administration induces inflammation in the ileum that is associated with enhanced expression of EpCAM, decreased claudin-4 expression and increased goblet cell density, these changes may contribute to systemic inflammation that is known to accompany many inflammatory diseases of the lung.

## Introduction

Inflammatory lung diseases (ILDs) encapsulate a collection of disorders affecting the airways and lungs such as chronic obstructive pulmonary disease (COPD), asthma, interstitial lung disease, acute lower respiratory tract infections and cystic fibrosis (CF) [1]. Collectively, these conditions (and more) affect more than 400 million people across the globe and contribute to the premature death of approximately 4 million people [2].

Many of these ILDs are often accompanied by co-morbidities, especially symptoms of gastrointestinal (GI) dysfunction [3–8]. Both adults and children with asthma suffer from gastrointestinal symptoms, particularly diarrhoea, vomiting, and abdominal pain, significantly more frequently than age-matched controls [9, 10], whilst gastroesophageal reflux disease (GERD) prevalence is higher in patients with interstitial lung disease [11]. Further to this, several population-based studies have found that patients suffering from COPD have a significantly higher risk of developing both ulcerative colitis (UC) and Crohn’s disease (CD) [12], with severity of COPD associated with an increased risk of inflammatory bowel disease (IBD) [13]. In addition, inflammation induced by respiratory infections can result in significant changes in gut function, as recently demonstrated in severe acute respiratory syndrome coronavirus 2 (SARS-CoV-2) virus disruption of intestinal barrier functions [14]. These and other data have resulted in the concept of bidirectional communication between the gut and lung, termed the Gut-Lung Axis (GLA).

To date, much of the research investigating the GLA in respiratory conditions has revolved around microbial dysbiosis [3, 15–18]. It has been shown that there is symbiotic alteration in the intestinal and respiratory microbiota in several ILDs [19–24], indicating that there is a vital crosstalk between these two mucosal sites of the human body. Intestinal microbiota and intestinal inflammation are associated with “leaky gut”, a state of pathogenic hyperpermeability in the GI tract. Leaky gut is thought to facilitate translocation of bacteria, bacterial metabolites, inflammatory cells and cytokines into systemic circulation allowing access to distant tissues which may contribute to remote organ injury [25].

Inflammatory or ulcerating diseases in the GI tract are known to be associated with abnormal intestinal barrier function [26]. Patients with celiac disease, IBD, and irritable bowel syndrome (IBS) all demonstrate abnormal intestinal permeability [27–29], with longitudinal studies indicating that increased intestinal permeability precedes relapse of Crohn’s disease suggesting a potential pathogenetic role of the epithelial barrier in GI inflammation [22, 26, 30]. Increased intestinal permeability has been shown in several metabolic diseases including obesity and diabetes, and most importantly, has been linked to increased systemic inflammation [22, 31–33].

In this study, we investigated and compared the effects of intratracheal LPS-induced lung inflammation at 24 and 72 h post LPS-administration on intestinal barrier integrity (Claudin-1, and EpCAM), intestinal inflammation (CD45 +), and correlated these findings with enteric neuropathy (Hu + cells/ganglia, proportion of neuronal nitric oxide synthase (nNOS) neurons, glial cell expression). We hypothesize that intratracheal LPS-induced lung inflammation will be associated with increased inflammation in the intestinal mucosa, reduced expression of tight junction markers, and changes at the level of the enteric nervous system (ENS).

## Materials and methods

### Mice

Female C57bl/6 mice aged 6–8 weeks (18–25 g) supplied from the Animal Resources Centre (Perth, Australia) were used for all experiments. Mice were allowed to acclimate for 1 week prior to experimentation, had free access to food and water and were kept under a 12-h light/dark cycle in a well-ventilated room at a temperature of 22 °C. All procedures were approved by The University of Melbourne Animal Experimentation Ethics Committee (#2020–10,077-2038–4) and performed in accordance with the guidelines of the National Health and Medical Research Council (NHMRC) *Australian Code of Practice for the Care and Use of Animals for Scientific Purposes.* A total of 32 animals were used in this study.

### Study design and intratracheal administration of LPS

Mice were assigned to treatment groups (*n* = 8 mice/group) based on housing configurations (LPS and vehicle control treated mice were not co-housed) and then anesthetized with an intraperitoneal injection of a ketamine/xylazine (90/10 mg/kg) solution. LPS (from *E.coli* 0111:B4 strain, 5 µg in 50 µl of sterile saline) (or vehicle control) was then administered intratracheally via a blunt polyethylene tube connected to a microsprayer (0.64 mm diameter; Penn Century, Pennsylvania, United States) attached to a high-pressure syringe to induce acute inflammation. To avoid false intubation into the oesophagus the trachea was illuminated via an otoscope held perpendicularly to the animal's throat, producing a well-illuminated trachea through dispersed light. Vehicle control and LPS-treated mice were anesthetized and treated alternately to minimise potential confounders.

### Cell and tissue collection

At 24 or 72 h after LPS treatment, mice were killed by an overdose of sodium pentobarbitone (intraperitoneal, 200 mg/kg). Vehicle control and LPS-treated mice were killed alternately to minimise potential confounders. Bronchoalveolar lavage fluid (BALF) was collected by inserting a catheter into the trachea and aspirating the airways once with 400 µl and 3 times with 300 µl aliquots of PBS. Total cell numbers were determined by visually counting cells using a glass hemocytometer. Cell differentials were assessed by adhering BALF cells to glass slides by cytospin, and staining with Diff- QuickTM (Dade A.G., Düdingen, Switzerland) as per the manufacturer’s instructions. Cells were identified as either macrophages/monocytes, neutrophils, or lymphocytes according to standard morphological criteria.

Segments of distal ileum and distal colon (3–5 cm) were dissected from the abdomen of each mouse and placed in oxygenated phosphate buffered saline (PBS; pH 7.2) for 10 min to inhibit smooth muscle contraction. They were then cut open along the mesenteric border, cleared of their contents, and pinned to balsa wood board mucosa-side up and fixed with Zamboni’s fixative (2% formaldehyde, 0.2% picric acid in PBS) overnight at 4 °C.

### Transepithelial resistance

Segments of distal ileum were opened along the mesenteric border and full-thickness segments (with the external muscle included) were pinned onto Ussing chamber sliders (P2311, 0.3 cm2 apertures, Physiological Instruments, San Diego, CA, USA) in physiological saline (Krebs’ solution: 11.1 mM glucose, 118 mM NaCl, 4.8 mM KCl, 1.0 mM NaH2PO4, 1.2 mM MgSO4, 25 mM NaHCO3, 2.5 mM CaCl2, pH 7.4). Sliders were inserted into two-part chambers (EasyMount Diffusion Chambers, Physiologic Instruments) and 5 mL Krebs’ solution was added to both sides, with the mucosal Krebs’ solution containing 11.1 mM mannitol instead of glucose. The glucose is required as an energy substrate but is replaced with mannitol in the mucosal solution to restrict active transport while maintaining osmotic balance. Solutions were kept at 37 ℃ and gassed with carbogen (5% CO2, 95% O2) to maintain pH. A multichannel voltage-current clamp (VCC MC6, Physiologic Instruments) was linked to each chamber through a set of four electrodes (2 voltage sensing and 2 current passing electrodes) and agar bridges (3% agarose/3 M KCl in the tip and backfilled with 3 M KCl) installed on opposite sides of the tissue. Voltage and Isc readings were acquired using a PowerLab amplifier and recorded using LabChart® 5 (both ADInstruments, Sydney, Australia).

Tissue was left to equilibrate for 20 min before clamping the voltage to 0 V. Transepithelial resistance (TER, Ω·cm2) was calculated by Ohm’s law multiplied by the surface area by administering 2 s pulses of 2 mV every 60 s throughout the course of the experiment.

### Gut histology

Ileum and colon preparations were cleared of fixative by washing 3 × 10 min with dimethyl sulphoxide (DMSO; Sigma-Aldrich, Australia) followed by 3 × 10 min washes with PBS. After washing, tissues were embedded in 100% OCT and frozen using liquid nitrogen (LN_2_) and isopentane (2-methyl butane) and stored in − 80 °C freezer. Tissues were cut at 20 μm section thickness using a Leica CM1950 cryostat (Leica Biosystems, Germany), adhered to slides and allowed to rest for 30 min at room temperature before processing. To examine goblet cells in the ileum and colon, Alcian Blue staining protocol was followed [34]. Intensity of goblet cell staining was quantified using image J software (NIH, MD, USA). All images were coded and analysed blindly.

### Lung histology

Lungs were dissected and processed as previously described [35]. Briefly, lungs were dissected out and placed in 10 × volume formalin overnight. The following day, all lungs were transferred in PBS for storage until required for sectioning. Fixed lungs were embedded in paraffin blocks for sectioning. Five-micron ribbon sections were cut using a Microm HM 325 paraffin microtome (Fisher Scientific) and placed in a 48 °C water bath prior to being gently adhered onto glass slides. The slides were incubated at 37 °C overnight. All slides were stained with hematoxylin and eosin (H&E) for morphological assessment. Images were captured with a Zeiss imaging microscope with the corresponding Zen software. A total of 12 lung samples were imaged, two from each experiment group, all capturing the entirety of the right lung for consistency.

### Western blot

Approximately 10-15 mg of snap frozen ileum and colon samples were homogenized in ice-cold RadioImmunoPrecipitation Assay (RIPA) lysis buffer (50 mM Tris–HCl, pH 7.4, 150 mM NaCl, 0.5% Sodium Deoxycholate, 1% Triton X-100, 0.1% SDS, 1 mM EDTA with protease/phosphatase inhibitors, 1 mM PMSF, 1 g/mL Aprotinin, 1 g/ml Leupeptin, 1 mM Benzamidine, 1 mM Na3VO4, 5 mM Na Pyrophosphate, 1 mM DTT and 1 mM NaF). Tissues were homogenized using an Omni Tissue Homogenizer (TH220, Omni International, Kennesaw, GA, USA) for 30 s followed by rotation for 1 h at 4 °C. Tissues were subsequently sonicated on ice for 30 s at a power of 90 watts in rounds of 10 s/10 s rest). Samples were centrifuged at 10,000×*g* for 10 min at 4 °C and the supernatant was transferred to a fresh microfuge tube without disturbing the pellet. Protein levels were quantified using the Pierce BCA Protein Assay Kit (Thermo Scientific) following the manufacturer’s protocol. Approximately 10ug of protein extracts were loaded onto a 26 well Criterion TGX Stain-Free gel (Bio-Rad Laboratories, Hercules, USA). The proteins were separated by electrophoresis (80 mV for 20 min, followed by 120 mV for 60–90 min). Once completed, the gel was removed and activated by exposure to UV light for 1 min, using the ChemiDoc MP system. Proteins were then transferred to nitrocellulose membrane blots, using the Trans-blot Turbo transfer system. Images of the membrane were then taken using the ChemiDoc MP system for total protein measurement. Membranes were then incubated for 1 h at room temperature with blocking buffer (5% skimmed milk). The membranes were then washed with tris-buffered saline with tween 20 (TBST) (3 × 10 min). The membranes were incubated with primary antibodies diluted at 1:1000 in 5% bovine serum albumin (BSA), 0.02% sodium azide and TBST, overnight at 4 °C. The primary antibodies were claudin-1 (rabbit, 1:100, Life Technologies, Cat#717,800, CA, USA), claudin-4 (rabbit; 1:500, Abcam, Cat#53,156, Cambridge, United Kingdom) and TLR4 (mouse; 1:500, Abcam, Cat#22,048, Cambridge, United Kingdom). Membranes were washed with TBST (3 × 10 min) and incubated with secondary antibodies diluted 1:10,000 in 5% skimmed milk and TBST for 1 h at room temperature. The membranes were then washed with TBST (3 × 10 min) before they were placed in Bio-Rad’s Clarity western ECL substrate (Biorad Laboratories, Hercules, USA) and imaged using the ChemiDoc™ MP system. Western blots were densitometrically analysed using Image Lab Software v5.2 (Bio-Rad). Protein content was then normalised to totally protein analysis by TGX stain-free gel (Bio-Rad Laboratories, Hercules, USA).

### Measurement of IL-6 in colon tissue

IL-6 levels were measured in snap frozen sections of colonic tissue using the Mouse IL-6 DuoSet ELISA kit (R&D Systems, MN, USA) according to the manufacturer's specifications. Samples were were homogenized as above, using an Omni Tissue Homogenizer in ice-cold RIPA lysis buffer. Protein levels were quantified using the Pierce BCA Protein Assay Kit (Thermo Scientific) following the manufacturer’s protocol. Approximately 400ug of protein extracts were diluted in RIPA and processed as per manufacturers specifications.

### Immunohistochemistry of intestinal cross section tissue

Ileum and colon preparations were cleared of fixative, embedded in OCT, and cut at 20 μm section thickness as described above.

Cross section preparations were incubated with 10% normal horse serum (Chemicon, CA, USA) for 1 h at room temperature. Tissues were then washed (2 × 5 min) with PBS and incubated with combinations of primary antibodies against CD45 (rat, 1:1000, BioLegend, Cat#147,702, CA, USA), EpCAM (rat, 1:1000, Abcam, Cat#187,276, Cambridge, United Kingdom), claudin-1 (rabbit, 1:100, Life Technologies, Cat#717,800, CA, USA), claudin-4 (rabbit; 1:500, Abcam, Cat#53,156, Cambridge, United Kingdom), GFAP (goat, 1:1000, Abcam, Cat#53,554, Cambridge, United Kingdom), and S100β (rabbit, 1:1000, Abcam, Cat#52,642, Cambridge, United Kingdom) overnight at 4 °C. All antibodies were commercially purchased with confirmed specificity through extensive validation. Sections were then washed in PBS (3 × 10 min) before incubation with the appropriate secondary antibodies labelled with fluorophore donkey anti-rat Alexa 594, donkey anti-chicken Alexa 488, donkey anti-rabbit, Alexa 488, donkey anti-goat Alexa 488, and donkey anti-rabbit Alexa 647 (all used at 1:500, Jackson Immunoresearch Laboratories, PA, USA) for 2 h at room temperature. The tissue was then washed (3 × 10 min in PBS) and incubated with Hoechst 33,258 solution (10 μg/ml Bisbenzimide-Blue in distilled water; Sigma-Aldrich, Sydney, NSW, Australia) for 5 min. The sections were given 3 × 10 min final washes in distilled H_2_O and then cover slipped using fluorescence mounting medium (DAKO, Australia).

### Immunohistochemistry of wholemount preparations

Wholemount preparations were incubated with 10% normal donkey serum (Chemicon, USA) for 1 h at room temperature. Tissues were then washed (2 × 5 min) with PBS and incubated with human anti-Hu (1:2000; a gift from Dr Miles Epstein) and sheep anti-nNOS (1:2000; a gift from Dr. Piers Emson) overnight at 4 °C. Tissues were then washed in PBS (3 × 10 min) before incubation with species-specific secondary antibodies labelled with different fluorophores: donkey anti-chicken Alexa 594 (1:200, Jackson Immunoresearch Laboratories, PA, USA) and donkey anti-goat Alexa 488 (1:200, Jackson Immunoresearch Laboratories, PA, USA) for 2 h at room temperature. The tissue was then washed (3 × 10 min in PBS) and incubated with Hoechst 33,258 solution (10 μg/ml Bisbenzimide-Blue in distilled water; Sigma-Aldrich, Sydney, NSW, Australia) for 5 min. Finally, the tissue was again washed before mounting onto glass slides and cover slipped using fluorescent mounting medium (Dako, Carpinteria, CA, USA).

### Image analysis

Prior to imaging, all slides were labeled by another researcher so that the experimenter was blinded to the treatment groups throughout imaging and analysis. The average number of Hu + neurons per area and proportion of nNOS + neurons were quantified in a minimum of 6 randomly chosen images per preparation (~ 200–400 neurons/image), from images captured with a 10 × air objective using the AxioImager.Z1 microscope (Carl Zeiss, Sydney, NSW, Australia) and processed using the multipoint tool in ImageJ software (NIH, MD). Ganglia, defined as clustered Hu + neurons less than 1 cell body width apart (~ 20 µm), were traced in image J software using the freehand tool. The number of Hu + neurons per ganglia was quantified using the multipoint tool.

Cross sectional immunohistochemistry was quantified in a minimum of 6 randomly chosen images per preparation, from images captured with either a 20 × air objective using an AxioImager.Z1 microscope (Carl Zeiss, Sydney, NSW, Australia) or a Nikon Eclipse Ti laser scanning microscope (for three dimensional (z-series) images; Nikon, Tokyo, Japan). All images were processed using ImageJ software (NIH, MD). Briefly, the mucosal or muscle region was traced using the freehand selection tool; the traced area was then duplicated, converted to 8-bit and thresholded to replicate the distribution of staining, and the analyse particles tool was used to calculate the mean grey value and percentage area of s100β, GFAP, claudin-1, EpCAM and CD45.

ImageJ software was also used to quantify Alcian Blue expression in cross sectional specimens. Images were deconvoluted to separate eosin and Alcian Blue channels, the analyse particles tool was then used to calculate the percentage area within a 75µmx75µm box placed over the mucosal region.

### Statistical analysis

Data were assessed using a Welch’s two-tailed t-test. Analyses were performed using Graph Pad Prism (Graph Pad Software Inc., CA, USA). Data are presented as mean ± standard error of the mean (SEM). Value differences were considered statistically significant at P < 0.05. Sample size was not predetermined by formal power statistical methods. Sample size was calculated based on our previous studies on enteric neuropathy and intestinal toxicity. The sample number of each experiment is presented in the figures. No animals were excluded from this study. Outliers in data sets were detected and excluded using Extreme Studentized Deviate test (Grubbs; alpha = 0.05) using Graph Pad Prism (Graph Pad Software Inc., CA, USA).

## Results

### Intratracheal LPS increases total cell numbers, particularly neutrophils, in BALF

**Fig. 1:**
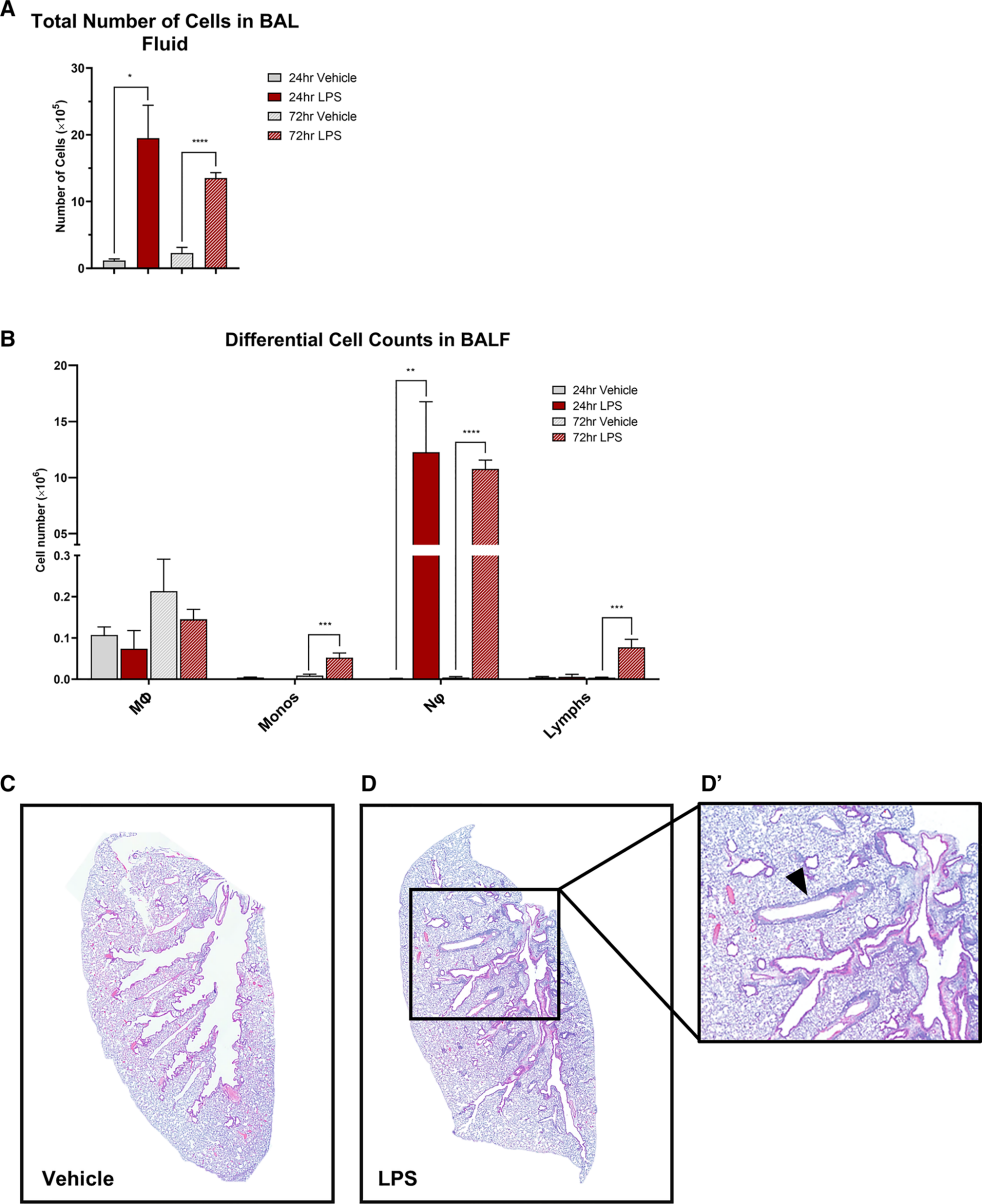
Increase in immune cells in BALF and lung following intratracheal LPS administration. Mice were treated with 5 μg of LPS and cell phenotype and numbers assessed in BALF. Total leukocyte (**A**) and leukocyte subset (**B**) cell numbers. Macrophages (MΦ), Monocytes (Monos), Neutrophils (Nφ), Lymphocytes (Lymphs). Representative images of lung H&E from vehicle (**C**) and LPS-treated (**D**, **D**’) lung. Data presented as mean ± SEM, and analysed using unpaired t-test with Welch’s correction. *P < 0.05, **P < 0.01, ***P < 0.001, ****P < 0.0001 significantly different to vehicle control, *n* = 4–8

Intratracheal administration of LPS induced a significant increase in total number of leukocytes in BALF at both 24 (*P* < 0.0001) and 72 (*P* < 0.007) hours compared to PBS controls (Fig. 1A). This was primarily driven by an initial influx of neutrophils at 24 h (P < 0.0003) which was maintained at 72 h (*P* < 0.000001, Fig. 1B). There was also a significant increase in monocytes (*P* < 0.0006) and lymphoytes (*P* < 0.0002) at 72 h compared to controls (Fig. 1B). Intratracheal administration of LPS induced application induced airway thickening and peribronchial immune cell infiltration, mild alveolitis and perivascular recruitment of inflammatory cells, indicative of local inflammation within the lung (Fig. 1D, D**`**).

### Intratracheal LPS increases the expression of CD45 + cells and goblet cells (alcian blue) selectively in the ileum

**Fig. 2:**
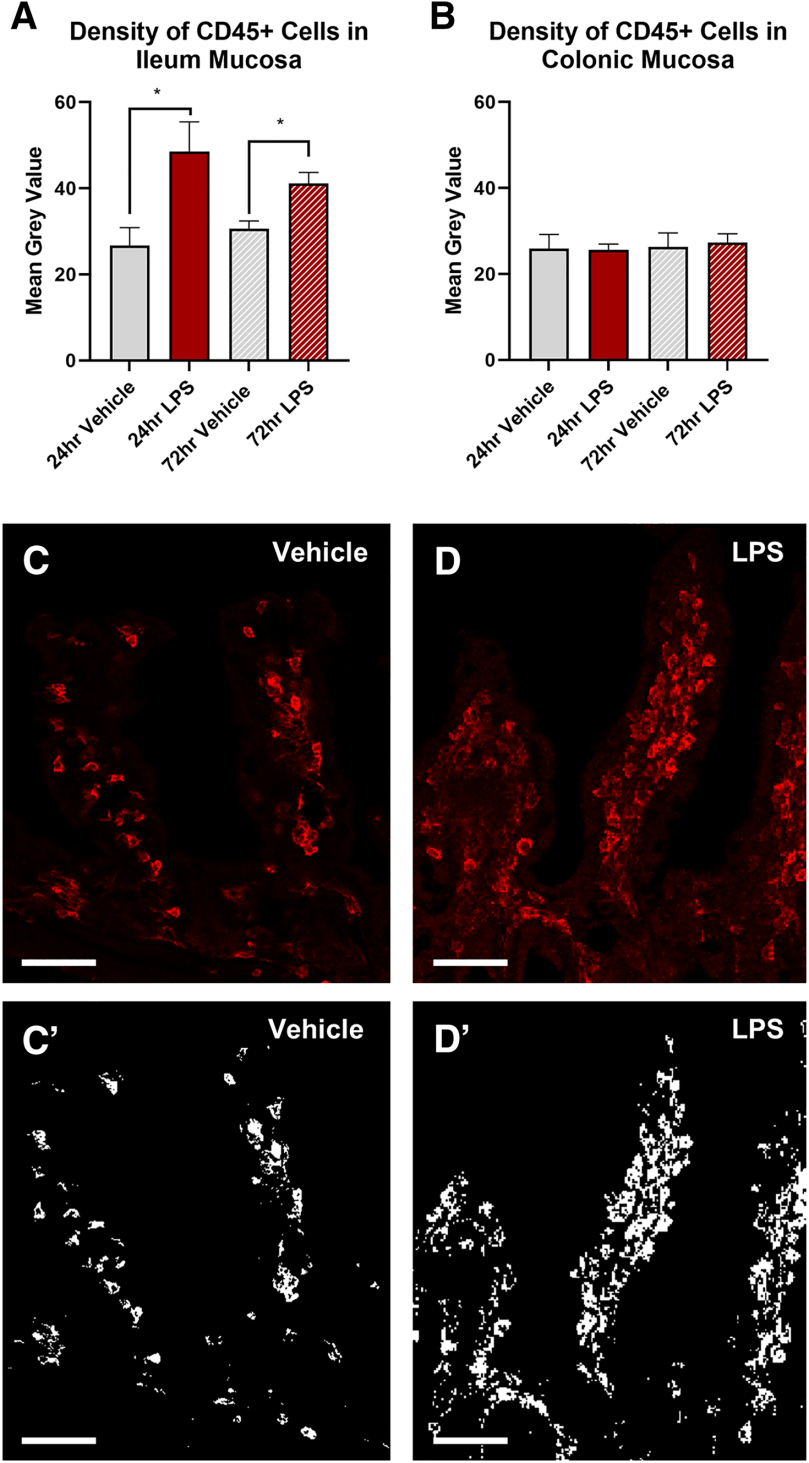
Density of CD45 + cells in the ileal and colonic mucosa following intratracheal LPS administration. CD45 + cells were significantly increased in the ileum at both 24 and 72 h following LPS administration (**A**). LPS had no effect on CD45 + cell expression in the colon (**B**). Representative photomicrographs of CD45 staining before and after thresholding in the ileum at 24 h following vehicle control (**C**,**C**`) and LPS (**D**, **D**`) administration. Scale bar = 50 µm. Data were analysed by unpaired t-test with Welch's correction. Data presented as mean ± SEM. *P < 0.05 significantly different to vehicle control, *n* = 3–7 for ileum data, n = 5 for colon data

**Fig. 3:**
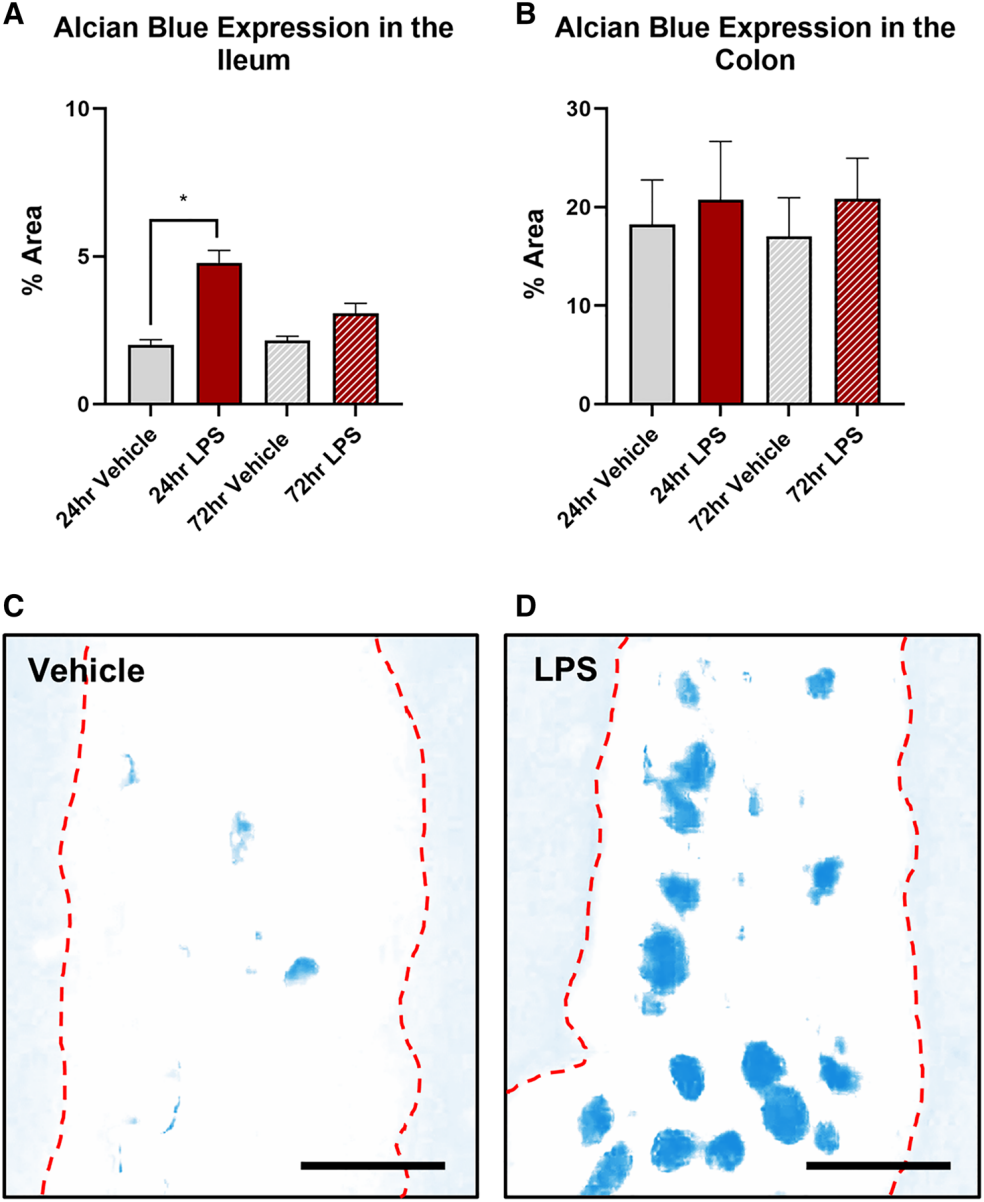
Effect on goblet cells numbers in the ileal and colonic mucosa following intratracheal LPS administration. Goblet cells were significantly increased in the ileum at 24 h following LPS administration compared to vehicle control (**A**). LPS had no effect on goblet cell in the colon (**B**). Representative photomicrographs of deconvoluted Alcian Blue staining in the ileum at 24 h following vehicle control (**C**) and LPS (**D**) administration. Scale bar = 25 µm. Data were analysed by unpaired *t*-test with Welch's correction. Data presented as mean ± SEM. *P < 0.05 significantly different to vehicle control, *n* = 3–5 for ileum and colon data.

Treatment with LPS significantly increased the density of CD45 + cells in the ileal mucosa at both 24 (vehicle: 26.7 ± 4.2 mean grey value; LPS: 48.6 ± 6.8 mean grey value, *P* < 0.05, Fig. 2A) and 72 h (vehicle: 30.7 ± 1.7 mean grey value; LPS: 41.2 ± 2.5 mean grey value, P < 0.05, Fig. 2A), however had no effect on the density of CD45 + cells in the colon (Fig. 2B). Alcian blue stining revealed that goblet cell number was transiently increased in the ileum at 24 h following LPS-treatment (vehicle: 2.0 ± 0.2% area; LPS: 4.8 ± 0.4% area, *P* < 0.05, Fig. 3A), however, was not significantly different at 72 h when compared to vehicle control (vehicle: 2.1 ± 0.1% area; LPS: 3.1 ± 0.3% area, Fig. 3A). Treatment with LPS had no effect on the number of goblet cells in the colon (Fig. 3B).

### Intratracheal LPS increases the expression of EpCAM in the ileum, but not the colon

**Fig. 4:**
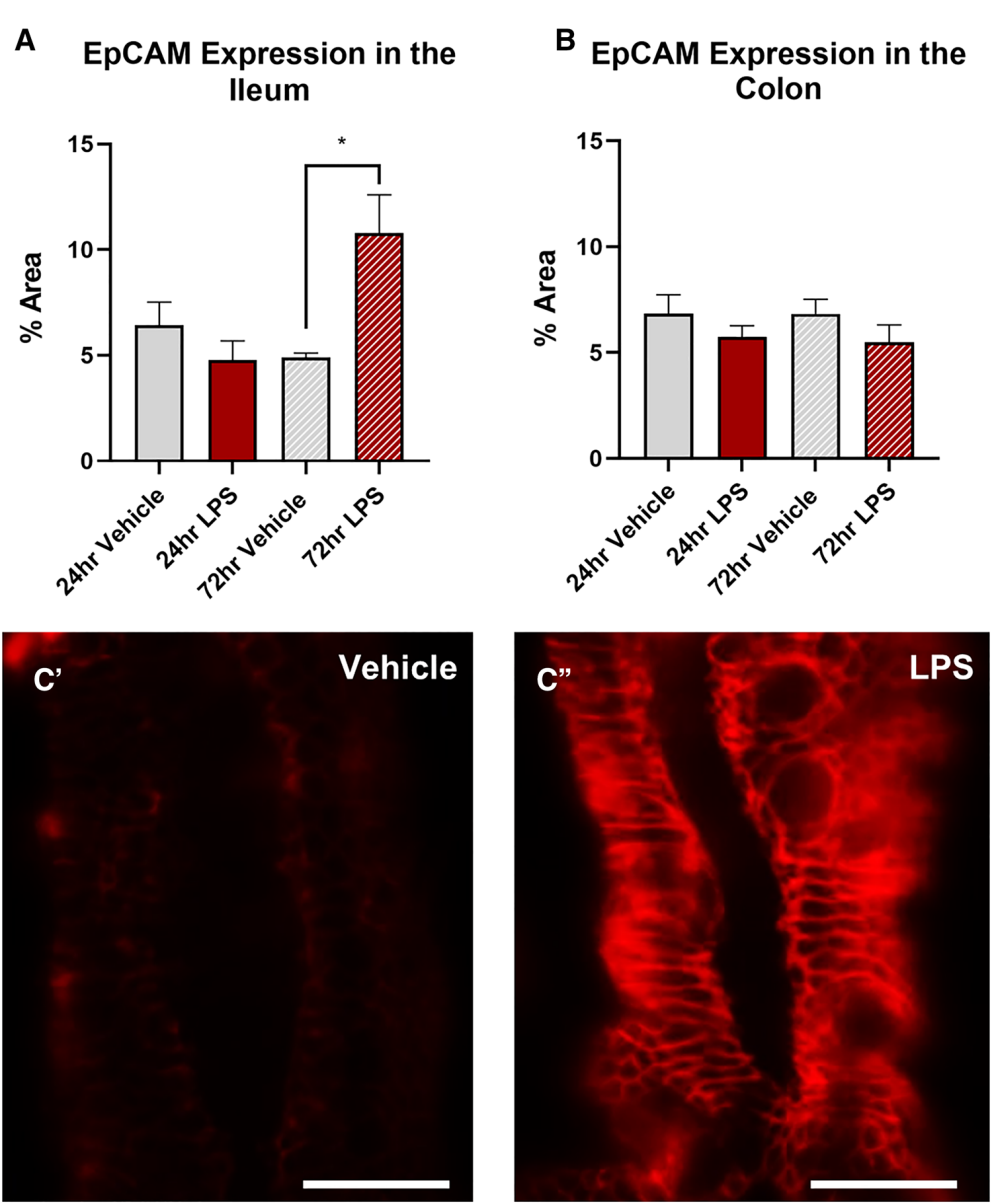
EpCAM expression in the ileal and colonic mucosa following intratracheal LPS administration. Percentage area of EpCAM expression in the mucosa was significantly increased in the ileum of mice at 72 h following LPS administration (**A**), however there was no effect in the colon (**B**). Representative photomicrographs of EpCAM staining in the ileum at 72 h following vehicle control (**A**`) and LPS (**B**`) administration, scale bar = 25 µm. Data were analysed by unpaired *t*-test with Welch's correction. Data presented as mean ± SEM. *P < 0.05 significantly different to vehicle control, *n* = 3–5 for ileum data, *n* = 4–6 for colon data

**Fig. 5:**
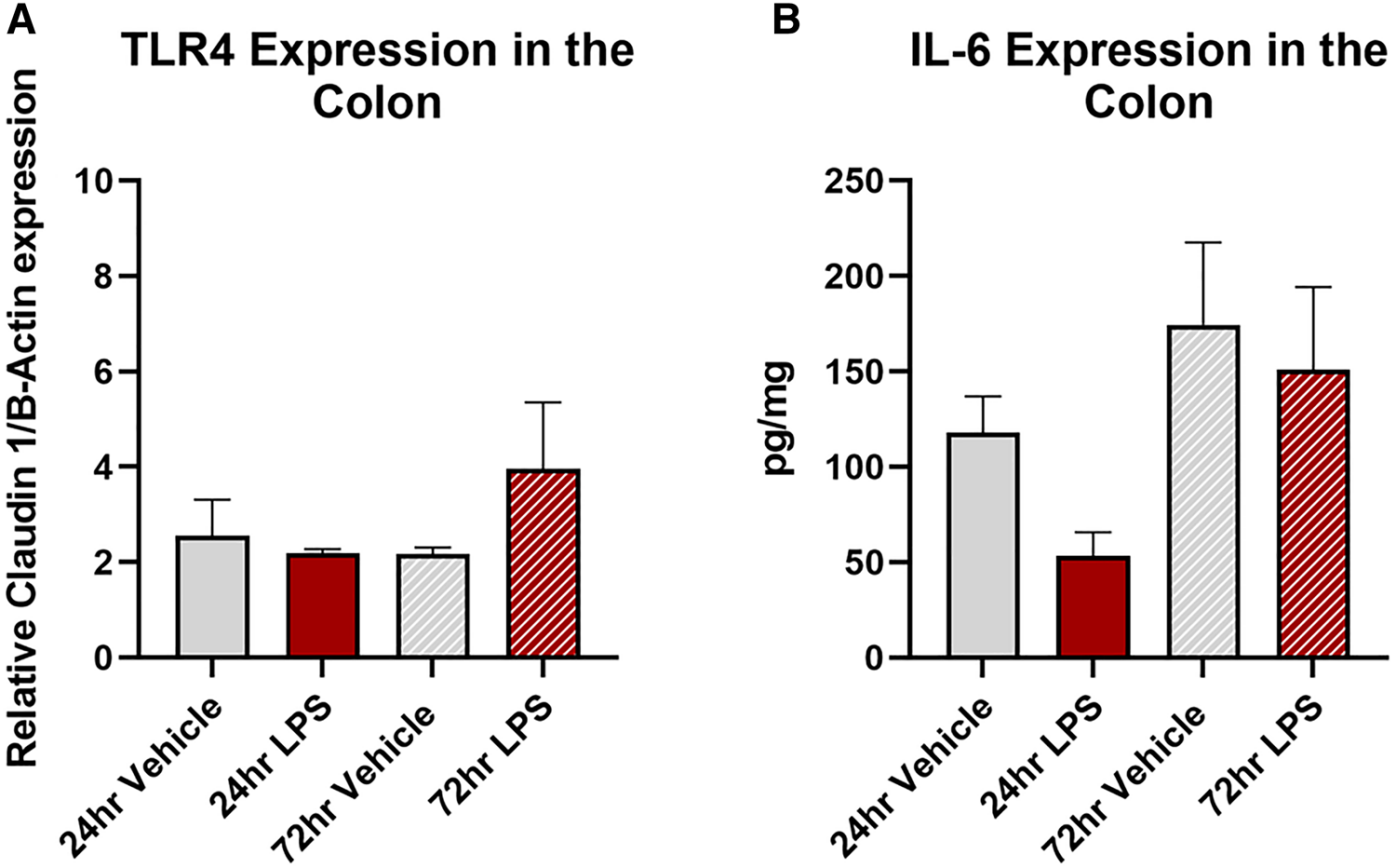
TLR4 and IL-6 expression in the colon following intratracheal LPS administration. Expression of TLR4 (**A**) and IL-6 (**B**) were unchanged in the colon at both 24 and 72-h following LPS-administration. Data were analysed by unpaired t-test with Welch's correction. Data presented as mean ± SEM

Treatment with LPS significantly increased the expression of EpCAM in the ileum at 72 h (vehicle: 4.9 ± 0.2%; LPS: 10.8 ± 1.8%, *P* < 0.05, Fig. 4A) but had no effect in the ileum at 24 h (vehicle: 6.4 ± 1.1%; LPS: 4.7 ± 0.9%, Fig. 4A), or in the colon at either 24 (vehicle: 6.8 ± 0.9%; LPS: 5.7 ± 0.7%, Fig. 4B) or 72 h (vehicle: 6.8 ± 0.8%; LPS: 5.5 ± 0.9%, Fig. 4B). Treatment with LPS had no effect on either TLR4 (Fig. 5A) or IL-6 (Fig. 5B) expression at 24 or 72 h in the colon.

### Intratracheal LPS decreased claudin-4 expression in the colon, but not the ileum

**Fig. 6:**
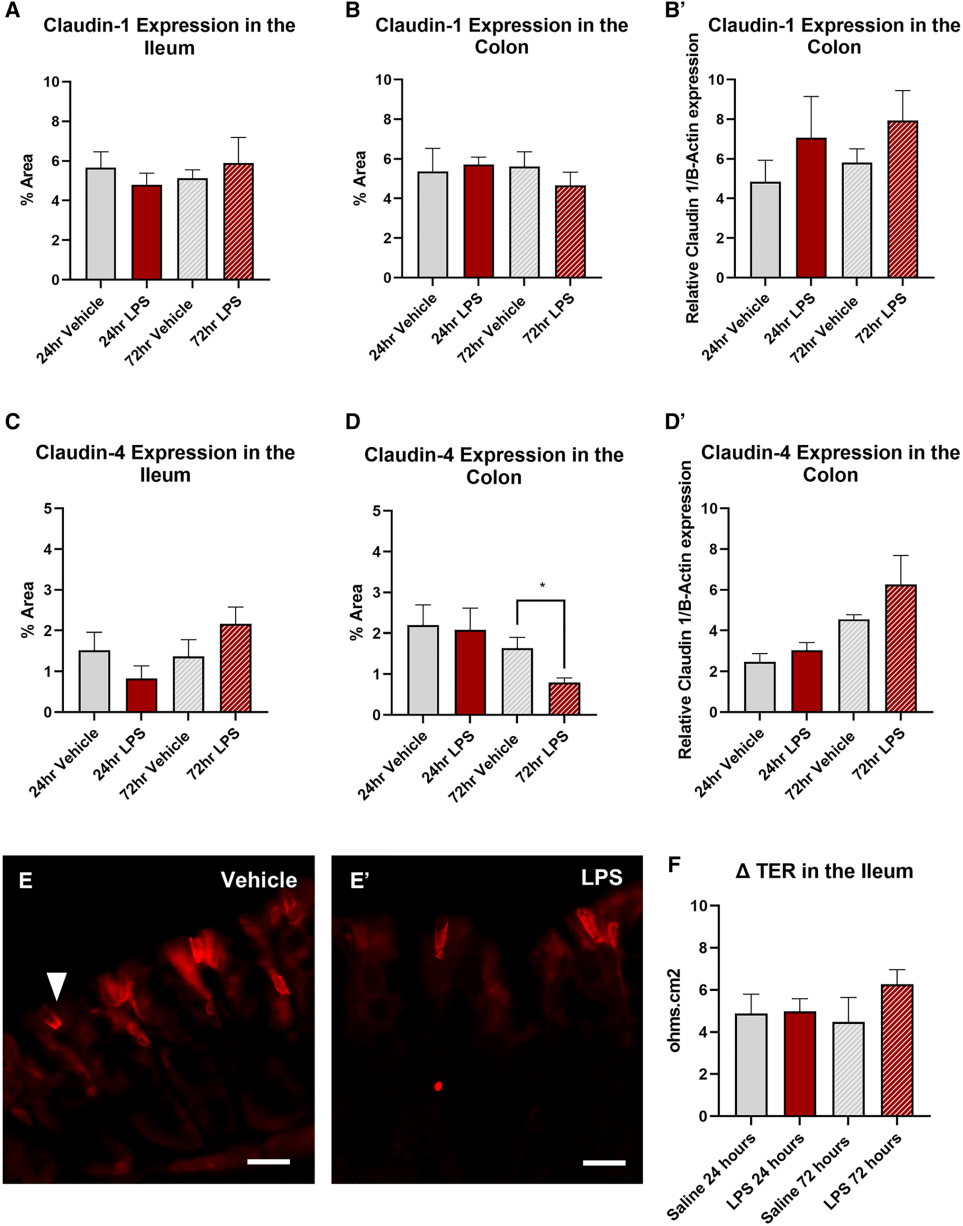
Claudin-1 and claudin-4 expression in the ileum and colon following intratracheal LPS administration. Percentage area of claudin-1 expression was unchanged in both the ileum (**A**) and colon (**B**) following LPS administration. Relative expression of claudin-1 in the colon was also unchanged at both 24 and 72-h following LPS-adminstration (**B**’). No significant difference in percentage area of claudin-4 expression was was found in the ileum (**C**), however a redction in claudin-4 expression was found in the colon at 72-h post LPS administration (**D**). Relative expression of claudin-4, as measured via western blot, was unchanged in the colon (**D**’). Representative images of claudin-4 staining in the colon from vehicle (**E**’) and LPS-treated mice (**E**’’). LPS treatment had no effect on transepithial resistance (TER) in the ileum (**F**). Data were analysed by unpaired t-test with Welch's correction. Data presented as mean ± SEM. *P < 0.05 significantly different to vehicle control, *n* = 3–6 for ileum data, *n* = 5–6 for colon data

Treatment with LPS had no effect on claudin-1 expression at either 24 or 72 h in the ileum (Fig. 6C) or colon (Fig. 6D, D`). However significantly reduced claudin-4 expression in the colon at 72 h (vehicle: 1.6 ± 0.3%: LPS: 0.8 ± 0.1%, *P* < 0.05, Fig. 6D, E–E`) but not the ileum at either 24 (vehicle: 1.5 ± 0.4%; LPS: 0.8 ± 0.3%, Fig. 6C) or 72 h (vehicle: 2.2 ± 0.4%; LPS: 1.4 ± 0.4%, Fig. 6C). Treatment with LPS had no effect on Δ transepithial resistance (TER) in the ileum at either 24 (vehicle: 4.9 ± 0.9 ΔTER; LPS: 5.0 ± 0.6 ΔTER, Fig. 6F) or 72 h (vehicle: 4.5 ± 1.2 ΔTER; LPS: 6.3 ± 0.7 ΔTER, Fig. 6F).

### Intratracheal LPS administration has no effect on enteric glial cells or enteric neurons in the ileum or colon

**Fig. 7:**
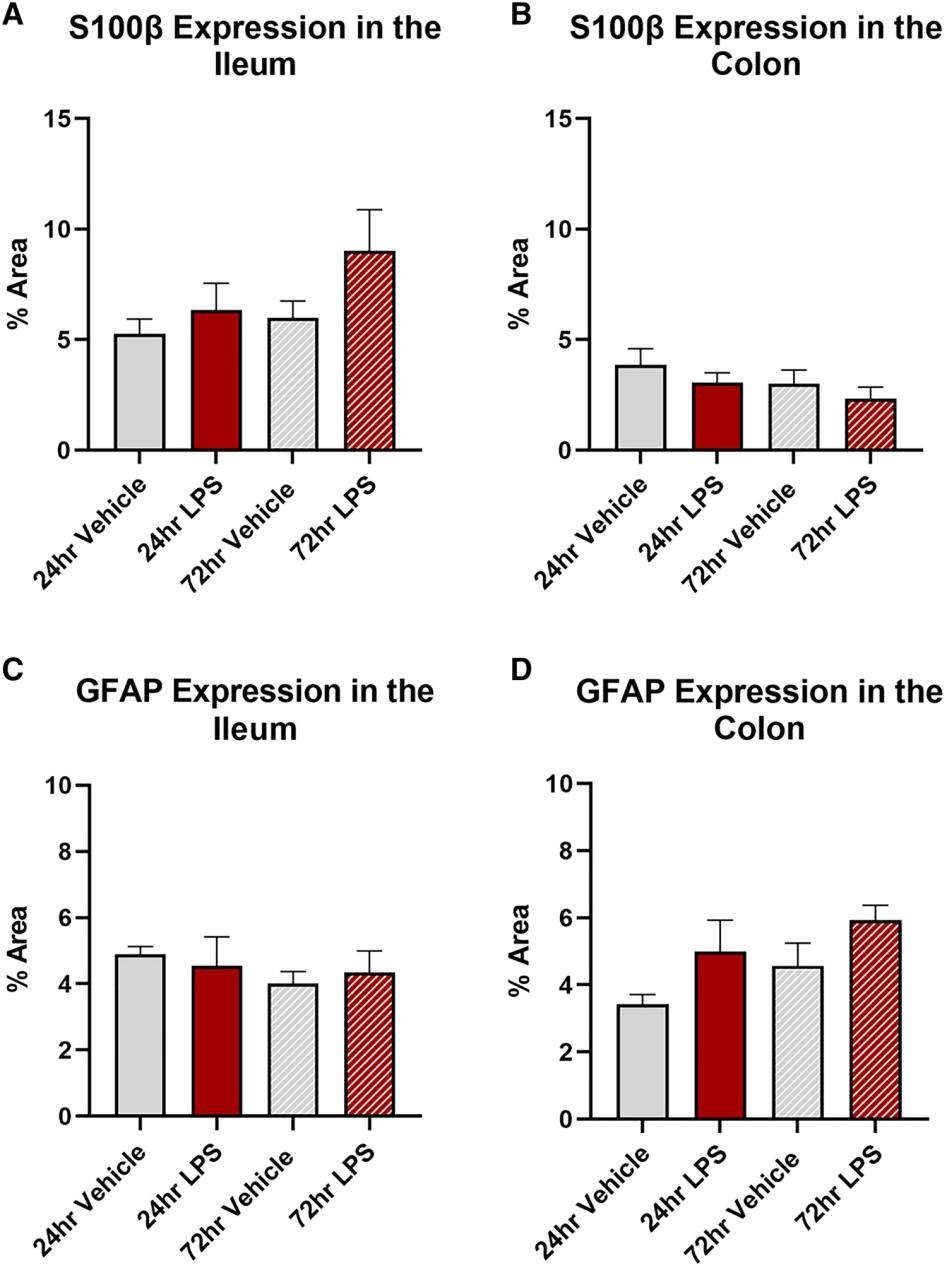
Glial cell expression in the ileum and colon following intratracheal LPS administration. Percentage area expressing S100β in the muscle layer of the ileum (**A**) and colon (**B**) was unchanged following LPS administration. Percentage area expressing GFAP in the muscle layer of the ileum (**C**) and colon (**D**) was unchanged following LPS administration Data were analysed by unpaired *t*-test with Welch's correction. Data presented as mean ± SEM, *n* = 3–7 for ileum data, *n* = 4–5 for colon data

Treatment with LPS had no significant effect on either S100β (Fig. 7A and 5B, Table 1) or GFAP (Fig. 7C,D, Table 1) expression at either 24 or 72 h in the ileum or colon.

**Table 1:** Summary of immunohistochemical changes in Claudin-1, S100β, GFAP and nNOS in the ileum and colon

	Ileum				Colon			
	24hr Vehicle	24hr LPS	72hr Vehicle	72hr LPS	24hr Vehicle	24hr LPS	72hr Vehicle	72hr LPS
Claudin-1%	5.7±0.8	4.7±0.6	5.1±0.4	5.9±1.2	5.4±1.1	5.7±0.4	5.6±0.7	4.7±0.7
S100β (%)	5.3±0.7	6.3±1.2	5.9±0.8	9.0±1.9	3.9±0.7	3.1±0.5	3.0±0.6	2.3±0.5
GFAP%	4.9±0.2	4.5±0.9	4.0±0.4	4.3±0.7	3.4±0.3	5.0±0.9	4.6±0.7	5.9±0.4
nNOS (%)	32.9±1.5	30.4±43.6	31.1±2.3	37.7±45.5	32.6±1.9	30.8±1.5	36.0±0.9	32.4±1.5

Data were analysed by unpaired t-test with Welch's correction. Data presented as mean ± SEM, n= 3-7 mice/group

**Fig. 8:**
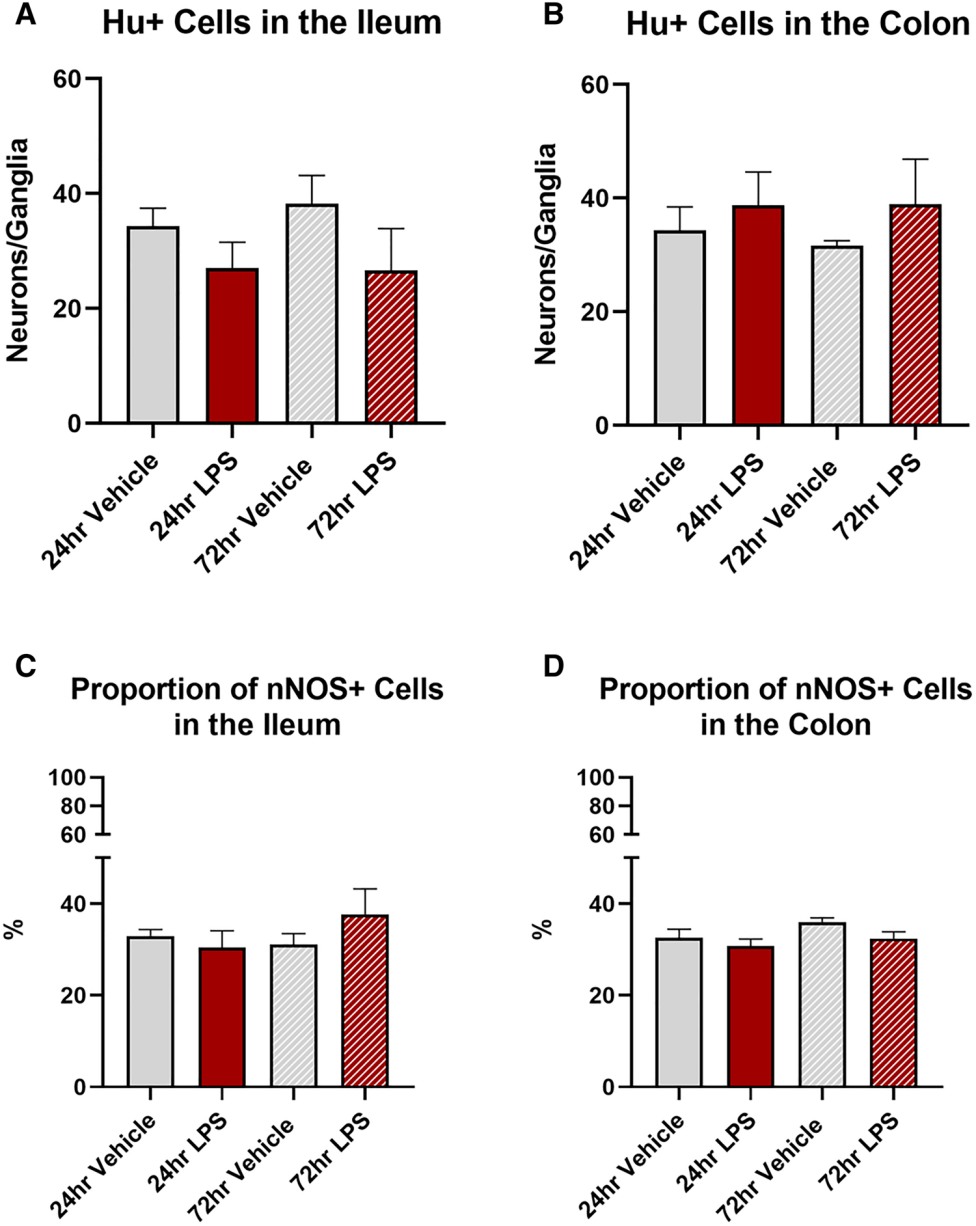
Number of Hu + neurons and proportion of nNOS + neurons in the myenteric plexus following intratracheal LPS. The number of Hu + myenteric neurons per ganglia was unchanged following LPS administration in both the ileum (**A**) and colon (**B**). Similarly, the proportion of nNOS neurons calculated to the total number of Hu + neurons was unchanged in both the ileum (**C**) and colon (**D**) following LPS administration. Data were analysed by unpaired *t*-test with Welch's correction. Data presented as mean ± SEM, *n* = 4–5 for ileum and colon data

Treatment with LPS had no significant effect on the number of myenteric neurons per ganglia at 24 h in either the ileum (vehicle: 34 ± 3.4 neurons/ganglia; LPS: 27 ± 4.5 neurons/ganglia, Fig. 8A) or colon (vehicle: 34 ± 4.1 neurons/ganglia; ileum LPS: 38 ± 5.9 neurons/ganglia Fig. 8B) or 72 h (ileum vehicle: 38 ± 4.9 neurons/ganglia; LPS: 26 ± 7.3 neurons/ganglia, colon vehicle 31 ± 0.8 neurons/ganglia; colon LPS: 38 ± 7.9 neurons/ganglia).

No significant difference in the proportion of nNOS-IR neurons was found in either the ileum (Fig. 8C, Table 1) or colon (Fig. 8D, Table 1) at 24 or 72 h.

## Discussion

ILD’s and inflammatory lung conditions due to respiratory infections are often associated with symptoms of GI dysfunction such as diarrhoea, vomiting, and abdominal pain. Though anecdotal evidence of gut manifestations in sufferers of respiratory disease has long been acknowledged, only recently has research begun to uncover the extent of gut-lung and lung-gut modulation in health and disease [3–8], resulting in the birth of the GLA concept. To date, the majority of research investigating the GLA has focused on microbial diversity and dysbiosis, with recent works highlighting the contribution of bacterial, fungal, and viral kingdoms at both digestive and respiratory levels [22, 36]. However, the effects of lung inflammation on other aspects of the GI tract, including functionality, remain largely overlooked. Several complex and interconnected systems/structures function to maintain intestinal barrier integrity, and indeed overall GI tract homeostasis, including the inflammatory system, ENS and mucosal barrier. The current study aimed to investigate the effect of LPS-induced lung inflammation on intestinal barrier integrity, enteric neurons, and mucosal inflammation. We demonstrated that localised lunginflammation generated by intratracheal administration of LPS induced intestinal inflammation, enhanced goblet cell numbers, and increased the expression of EpCAM in the ileum, whilst having little effect on the colon. Whilst these changes were not associated with any significant differences in number of neurons per ganglia or glial cell expression, when considered proportionally, LPS treated mice had 21% and 30% fewer neurons at 24 and 72 h respectively in the ileum, when compared to vehicle treated controls.

Our data indicate that lung inflammation is associated with enhanced inflammation in the ileum. LPS-treated mice displayed a significantly higher density of CD45 + cells at both 24 and 72 h in the ileum, but not the colon. Whilst this is the first study to our knowledge to demonstrate that acute inflammation in the lung can induce inflammation in the gut, there is known to be a strong association between chronic inflammatory lung conditions, such as COPD and asthma, and inflammatory diseases of the intestine, such as IBD and IBS [12, 37–39]. At an epidemiological level COPD patients have a significantly higher risk of both ulcerative colitis and Crohn’s disease [12, 40], and IBS patients exhibit a significantly higher risk of developing COPD compared with non-IBS patients [41]. Similarly, an association between asthma and IBD has been thoroughly shown [40, 42], with pre-existing asthma associated with an increased risk of developing Crohn’s disease or ulcerative colitis in later life [43]. Interestingly, our data demonstrated no significant difference in IL-6 expression in the colon. IL-6 plays a key anti-inflammatory role in acute phase inflammatory response and has been found to exert critical control over proinflammatory cytokines in models of endotoxic lung and endotoxemia [44]. Xing and colleagues demonstrated a peak in IL-6 expression in BALF at 4-h post LPS followed by a gradual declined over the following 20 h. Therefore, it is possible that lack of change in intestinal IL-6 following intracheal LPS in the current study may be due to timing of cull and tissues collection, or perhaps immune profile within the intestine does not directly mirror that of the lung. Further studies are required to investigate whether a transient increase in IL-6 expression occurs within the intestine at earlier (< 24 h) or later time points (> 72 h) following LPS-adminitration.

Whilst there is a clear relationship between inflammatory conditions of the gut and lung, whether or not lung inflammation is causative of gut inflammation, and vice versa, or whether the frequent co-occurrence of inflammatory lung and gut diseases results from shared genetic, environmental, and microbial risk factors remains poorly understood. Microbiota are known to closely interact with the mucosal immune system and the ENS within the GI tract [45–48], and has previously been shown to play a role in the modulation of gut function and immune response in conditions of GI dysfunction [49–52].

Further investigation is also required to determine what might underly the differential inflammatory responses in the small and large intestines observed in the current study. Literature surrounding inflammation in the small intestine, termed ileitis, in ILDs is sparse, however, in certain infectious lung conditions, such as histoplasmosis, propagation to the GI tract occurs via the reticuloendothelial system (RES) through macrophage accumulation in lymphoid aggregates and Peyer’s patches [53]. Given that Peyer’s patches are most densely concentrated in the distal ileum, this may explain why the ileum contains a higher density of inflammatory cells following intratracheal LPS administration [54].

Loss-of-barrier function as a result of mucosal inflammation contributes to the chronic nature of both respiratory and gastrointestinal conditions, although it is not yet understood whether loss of barrier function is a contributing/causative factor or a consequence of disease [12]. The mucosal layer plays a key role in maintaining intestinal barrier integrity. Goblet cells which reside throughout the length of the GI tract are responsible for synthesizing and secreting high-molecular-weight glycoproteins such as mucins which form a protective layer that lines the mucosa. The major component of GI mucus the MUC2 mucin [55]. In conditions of intestinal inflammation, mucin productions is known to be altered. Our data shows that intratracheal LPS administration and mucosal inflammation in the ileum is associated with a transient increase in goblet cell/mucin expression, whilst little effect was seen in the colon. This is in line with previous works which have shown that repeated delivery of LPS directly to the gut increases goblet cell number in the ileum [56], a response which is thought to be modulated by activation of NFκB and subsequent upregulation of MUC2 transcription [57]. Furthermore, deletion of MUC2 in the intestine has been found to induce abnormal intestinal morphology through increased mucosal thickness, flattening and ulceration of epithelial cells and an increase in inflammatory cell infiltration, indicating that goblet cell production of MUC2 is critical to the maintenance of the intestinal epithelial barrier structure and function [58].

Whilst intestinal goblet cell/MUC2 expression is yet to be investigated in ILDs, goblet cell hyperplasia in the lung is a common feature in both asthma and COPD [59, 60]. Changes in MUC2 expression have also been linked with increased intestinal permeability and morphological defects in intercellular tight junctions, with MUC2 knockout associated with increased permeability to FITC dextran and delocalization of tight junction protein Claudin-3 from the membrane [61]. This is in line with observations from IBS and IBD patients, who demonstrate downregulation of “sealing” tight junction proteins, and upregulation of pore forming tight junction molecules [62, 63].

To assess whether heightened inflammation and mucous production in the small intestine was associated with changes in indicators of tight junction function, we assessed ileal and colonic cross sections immunohistochemically using antibodies against EpCAM, claudin-1 and claudin-4. Our data indicate a significant increase in EpCAM + cells in the ileal mucosa at 72 h, however no change was detected in the colon. Enhanced EpCAM expression has been reported in other inflammatory conditions of the GI tract, however the majority of studies have focused on the colon rather than the small intestine [64].

A significant increase in the expression of EpCAM cells has been reported in the mucosa of paediatric patients with UC when compared to healthy controls and CD patients [64]. It has been suggested that enhanced epithelial cell expression could play a role in and/or be a result of intestinal epithelium regeneration [65], whilst extracellular vesicles from EpCAM + intestinal epithelial cells have been shown to participate in maintaining the intestinal tract immune balance [66]. The enhanced expression of EpCAM in the current study supports previous work suggesting that the intestinal epithelium plays a strategic role in GI inflammation, actively contributing to the activation and proliferation of different cellular components of the mucosal immune system as well as providing a physical barrier. Lack of change in EpCAM expression in the colon, which does not align with previous studies, may be due to the acute delivery and assessment of LPS-induced inflammation. UC and CD are chronic inflammatory conditions, which wax and wane over the lifetime. It is possible that changes in EpCAM and indeed inflammation, mucous production, and other TJ/cell adhesion molecules (CAMs) are delayed in the colon, occurring at a later stage in disease, which was unable to be detected by the current studies design. Further investigations into the involvement of peripheral and local cytokines is also required to better understand the balance between proinflammatory cytokines and anti-inflammatory molecules which undoubtably play a role in the severity and duration of mucosal inflammation.

Given that EpCAM is essential for cell junctions and interacts with several important cell adhesion molecules (CAMs) to regulate adhesive structures between cells [67], alteration in EpCAM expression might be expected to have an effect on on intestinal permeability. Whilst this assumption is supported by is evidence of GI leakiness in several ILDs as well as in LPS models, results from the current study found no change in ileal TER. Previous works have found that intraperitoneal LPS administration triggers an increase in intestinal TJ permeability both in vitro and in vivo, mediated by an increase in enterocyte membrane TLR-4 expression and a TLR-4-dependent increase in membrane colocalization of membrane-associated protein CD14 [68]. Similarly, intestinal permeability has been found to be increased in adults and children with asthma [69, 70], whilst intestinal hyperpermeability and impaired intestinal nutrient absorption has been reported in patients with COPD [71, 72]. Furthermore, in a study investigating GI consequences of ventilator-induced lung injury, GI index of permeability was significantly increased following lung ventilation and antagonism of tumour necrosis factor (TNF) with neutralizing antibodies abrogated this effect [73], highlighting the relevance of the lung-induced inflammatory cascade on remote organs, such as the gut. Though our TER results may insinuate that intratracheal LPS has no effect on intestinal permeability, it is worth noting that only a small area of one specific region of intestinal tissue was assessed. A limitation in this study and indeed with this technique is the small assessment area relative to intestinal length. The data presented in the current study reflects an assessment area of 0.3cm^2^ of distal ileum, whilst it is estimated that mean length of the mouse intestine (including duodenum, jejunum, ileum and colon) is ~ 54 cm [74]. Thus, further studies which adequately map changes in TER throughout different regions of the intestine (duodenum, jejunum, ileum and colon), or in vivo assessment of intestinal permeability to fluorescently tagged sugars, such as 4 kDa FITC-dextran are required to determine whether the intratracheal LPS-induced changes in EpCAM, claudin-4 and mucin have any functional effect on intestinal barrier function. Future studies should also explore other TJ/CAM markers such as ZO-1, occludin and other claudins (2, 3, 5, 7, 8, 12 and 18), all of which have been implicated in dysregulation of intestinal permeability in inflammatory conditions of the GI tract [75], and investigate markers of systemic inflammation, a known risk factor for systemic manifestations of disease in ILDs [76].

Whilst previous works have demonstrated the role of TLR4 in LPS-induced intestinal hyperpermeability [68], the current study found no significant difference in TLR4 expression in the colon folllwing intratracheal LPS administration. A likely explanation for this divergence in results is the early time-frame in which our samples were collected. Guo and colleagues report that intraperitoneal LPS treatment did not affect TLR-4 expression up to day 3; however, following 5 days of LPS treatment there was a marked increase in TLR-4 expression in the plasma [68].

Inflammation in the GI tract has been linked to damage and/or death of enteric neurons and alteration in expression of neuropeptides in a variety of conditions including Chagas Disease, UC, CD and IBS [77]. These changes at the level of the ENS are believed to contribute to/underly the GI dysfunction associated with these conditions. Whilst ILDs are known to be associated with GI dysfunction, the role of the ENS is yet to be investigated [3–8].

Our data indicate that LPS-induced lung inflammation has no effect on the number of myenteric neurons per ganglia. Interestingly, our data also indicate no change in the expression of glial cell markers GFAP and S100β. Previous studies have shown that enteric glial cells may contribute to chronic mucosal inflammation in patients with IBD [78, 79]. GFAP expression has been found to be altered in the mucosa of patients with ulcerative colitis (UC) and Crohn’s disease [79, 80]. Similarly, S100β immunoreactivity is significantly increased in the rectal mucosa of UC patients when compared to healthy controls [78]. Lack of change in glial cell markers in the current study may be due to the acute delivery of LPS and short term nature of the study. As mentioned previously, UC and CD are chronic inflammatory conditions, thus plastic changes in enteric glial cells may take several days, weeks or months to manifest.

## Conclusions

In conclusion, the data presented here, taken together with large scale epidemiological studies, demonstrate there may be a potential link between inflammatory diseases in the respiratory and intestinal systems. Our data indicate the intratracheal LPS administration is associated with inflammation, enhanced goblet cell and EpCAM expression in the ileum and reduced claudin-4 expression in the colon Both COPD and asthma are associated with systemic inflammation, given there is a known link between intestinal inflammation, intestinal barrier dysfunction, and systemic inflammation, it is possible that changes in the ileum contribute to low-grade systemic inflammation associated with inflammatory lung conditions.

Further research is required to determine how signals are transferred from the lung to the gut, the duration of intestinal inflammation, the functional consequences of intestinal inflammation and altered EpCAM expression on intestinal permeability/leakiness and whether these changes are associated with systemic inflammation.

## Abbreviations

BAL: Bronchoalveolar lavage
BALF: Bronchoalveolar lavage fluid
COPD: Chronic obstructive pulmonary disease
CD: Crohn’s disease
CF: Cystic fibrosis
ILD: Inflamatory lung disease
DMSO: Dimethyl sulfoxide
ENS: Enteric nervous system
GI: Gastrointestinal
IBD: Inflammatory bowel disease
IR: Immunoreactive
LPS: Lipopolysaccharide
nNOS: Neuronal nitric oxide synthase
PBS: Phosphate buffered saline
UC: Ulcerative colitis
